# Determinants of compliance with malaria chemoprophylaxis among French soldiers during missions in inter-tropical Africa

**DOI:** 10.1186/1475-2875-9-41

**Published:** 2010-02-03

**Authors:** Noémie Resseguier, Vanessa Machault, Lénaick Ollivier, Eve Orlandi-Pradines, Gaetan Texier, Bruno Pradines, Jean Gaudart, Alain Buguet, Catherine Tourette-Turgis, Christophe Rogier

**Affiliations:** 1 Institute for Biomedical Research of the French Army (IRBA) & URMITE UMR6236, Allée du Médecin Colonel Jamot, Parc du Pharo, BP60109, 13262 Marseille cedex 07, France; 2 Aix Marseille University, Faculty of Medicine Marseille, Laboratory of Education and Research in Medical Information Processing (LERTIM) EA 3283, Biostatistics Research Unit, Marseille, France; 3 Assistance Publique - Hôpitaux de Marseille, SSPIM Timone, Marseille, France; 4 Département d'épidémiologie et de santé publique & EA3283, Parc du Pharo, 13262 Marseille cedex 07, France; 5 EA4170 Free Radicals, Energy Substrates and Physiopathology, Claude-Bernard Lyon I University, 8 Avenue Rockefeller, 69373 Lyon cedex 08, France; 6 Institut d'éducation thérapeutique, Fondation partenariale, University Pierre et Marie Curie, Paris 6, and University of Rouen, France

## Abstract

**Background:**

The effectiveness of malaria chemoprophylaxis is limited by the lack of compliance whose determinants are not well known.

**Methods:**

The compliance with malaria chemoprophylaxis has been estimated and analysed by validated questionnaires administered before and after the short-term missions (about four months) in five tropical African countries of 2,093 French soldiers from 19 military companies involved in a prospective cohort study. "Correct compliance" was defined as "no missed doses" of daily drug intake during the entire mission and was analysed using multiple mixed-effect logistic regression model.

**Results:**

The averaged prevalence rate of correct compliance was 46.2%, ranging from 9.6%to 76.6% according to the companies. Incorrect compliance was significantly associated with eveningness (p = 0.028), a medical history of clinical malaria (p < 0.001) and a perceived mosquito attractiveness inferior or superior to the others (p < 0.007). Correct compliance was significantly associated with the systematic use of protective measures against mosquito bites (p < 0.001), the type of military operations (combat vs. training activities, p < 0.001) and other individual factors (p < 0.05).

**Conclusions:**

The identification of circumstances and profiles of persons at higher risk of lack of compliance would pave the way to specifically targeted strategies aimed to improve compliance with malaria chemoprophylaxis and, therefore, its effectiveness.

## Background

Non-immune civilians and military personnel traveling in malaria-endemic areas are at risk of getting malaria and may become clinically ill during or after their travel. Approximately 25-30 million travellers from non-tropical regions visit malaria-endemic countries annually, and about 30,000 cases of travel-associated clinical malaria occur each year [1]. In the UK, the incidence of imported clinical malaria among civilian travellers visiting West Africa varied from 196 cases to 52 cases/1,000 traveller-years between 2003 and 2006 [2]. In a Swedish survey, conducted from 1997 to 2003, the annual incidence rate of clinical malaria was 240, 302 and 357 per 100,000 travellers to East Africa, West Africa and Central Africa, respectively [3]. In a cohort of the French general population, followed from 1994 to 1998, the incidence of malaria imported from endemic areas was 435 cases per 100,000 trips, corresponding to 178 cases per 1,000 traveller-years [4]. In the French Armed Forces, the annual incidence rate was 14 per 1,000 person-years in 2006, and half of the 558 cases occurred after the patients returned to France. Among French soldiers serving in Côte d'Ivoire between 1998 and 2006, the annual malaria incidence rate ranged from 27.5 to 294.7 cases per 1,000 person-years; the maximum rate resulted from an epidemic among the troops during fighting operations in 2002. The risk of malaria for travellers varies notably between endemic areas and periods of exposure [5].

Malaria is an important threat to tourists, soldiers and employees travelling or working in endemic areas, particularly because of the potentially rapid onset of infection and the severity of the disease. Non-immune travellers should be protected from malaria by chemoprophylaxis and prophylactic measures against mosquito bites, including insecticide-impregnated bed nets (IIBN), repellents and insecticide-treated long-sleeved clothes and pants. In malaria-endemic areas, the use of most of these prophylactic measures is mandatory for non-immune soldiers of Western armies or for non-immune employees of most major international groups. The resistance of *Plasmodium falciparum *to most anti-malarial drugs and the resistance of *Anopheles *to insecticides may limit the efficacy of these prophylactic measures. Chemoprophylaxis is a key component of malaria prevention because none of the vector protection measures totally avoid mosquito bites during night activities. However, the effectiveness of chemoprophylaxis is limited by lack of compliance with drug intake, even if the regimen is adapted to the chemosusceptibility of *P. falciparum *[6,7].

Many clinical malaria cases and outbreaks [8-11] have been attributed to suboptimal compliance with chemoprophylaxis [12,13], including among military personnel [14-16]. In a cohort study, lack of compliance with prophylactic measures was the second most important factor that determined the malaria incidence rate among non-immune travellers [5].

It is generally acknowledged that the lack of compliance with medications is unrelated to the lack of symptoms or risk perception. In fact, compliance determinants are not well known, especially for chronic diseases, such as HIV infection [17,18], diabetes [19,20], psychosis [21,22], tuberculosis [23,24], or for malaria chemoprophylaxis [25-28]. A better understanding of the determinants of the compliance with malaria chemoprophylaxis could pave the way for the improvement of its effectiveness and for the development of interventions aimed at enhancing compliance.

The objective of the present study was to identify the determinants of compliance with malaria chemoprophylaxis among French soldiers on a short-term mission in malaria-endemic areas of tropical Africa.

## Methods

### Study design and inclusion criteria

The present study was part of a larger prospective cohort study on the risks of vector-borne diseases among French soldiers carried out between February 2004 and September 2007. The study comprised 19 French military companies (100 to 150 individuals per company) serving a short-term mission (about four months) in five tropical African countries: eight companies in Côte d'Ivoire, four in Gabon, three in Chad, two in Central African Republic and two in Senegal. The soldiers were required to take chemoprophylaxis and apply protective measures against mosquitoes. These companies were selected to represent the various conditions that French soldiers experience in Africa. All members of the companies designated for the mission were eligible for the study and were invited to participate. Soldiers absent from the regiment on the dates of the surveys or who left the company between the survey and the departure or during the mission were excluded from the analysis.

The protocol was approved by the ethical committee Marseille II (Advice no. 02/81, 12/13/2002). The informed consent of each participant was obtained at the beginning of the study after a thorough explanation of its purpose.

Two self-administered questionnaires containing behavioural items were filled out by each soldier and validated by a member of the research team. The first survey was administered no earlier than 15 days before their departure, and the second was completed no later than 15 days after their return.

### Data collection

The dependant variable was the level of compliance with malaria chemoprophylaxis during the mission as assessed retrospectively by the return questionnaire. "Correct" compliance was defined as "No missed doses" of daily drug intake during the entire mission, while "incorrect" compliance was defined as one of the three other possible responses: "Less than one missed dose per month," "One or more missed doses per month, but less than one missed dose per week" or "One or more missed doses per week".

Independent variables that were considered potential determinants of compliance were recorded for each soldier either upon departure or return. These variables were related to individual characteristics or to the particular settings of the mission, as described below.

Variables relative to the mission were the dates of departure and return, country and mission type, such asmilitary operation or training. Demographic and behavioural variables were age, gender, rank, usual morningness and eveningness (as determined using the reduced Horne and Östberg morningness-eveningness questionnaire [29-31]), wake and sleep times during the mission, previous travel overseas or other malaria-endemic areas and tobacco consumption. Morningness and eveningness were used to determine the chronotype, *i.e. *the circadian rhythm of the soldiers: morning people waking up early and being more alert in the first part of the day, evening people being more alert in the late evening hours and preferring to go to bed late.

Other variables pertained to the occurrence of important familial events during the mission (*e.g.*, birth of a child, illness or death of a relative or friend, or separation or announcement of a divorce). Other variables included the subjective perceived risk level during the mission associated with i) a dangerous situation, ii) the individual likelihood of acquiring clinical malaria, and iii) the severity of disease if malaria were contracted.

Variables related to protective measures against vectors included the condition of the received IIBN and the use of each protective measure designated for each type of activity during the mission (guard, operation out of the military camp, free time, and time in the military camp). A global score was calculated by combining the frequency of use of each antivectorial measure during each activity with the time spent in each activity. Moreover, a global variable summarizing the use of all anti-vectorial protective measures (including repellents, long sleeved clothes and IIBN) was constructed.

Independent variables relative to the chemoprophylaxis were the drug itself (either doxycycline or chloroquine-proguanil combination with once-daily dosages), tolerance and usual time schedules of intake.

The variables incorporating medical antecedents were allergy to insect bites, usual cutaneous response to mosquito bites and medical history of malaria, leishmaniasis or dengue fever (*i.e.*, themain vector-borne diseases experienced by the French Forces). Variables pertaining to medical events that occurred during the mission included clinical malaria, non-malarial fever, diarrhoea, boil, other severe infectious disease (*i.e.*, medical issues that required hospitalization or drug treatment for one month or more), perceived frequency of mosquito bites and perceived personal mosquito attractiveness.

### Statistical methods

Data were recorded using EpiDATA v3.0 and were checked for consistency before statistical analysis using STATA 9.0 (StataCorpLP, College Station, TX, USA). Only participants who completed both questionnaires were considered for statistical analysis. Missing values affected less than 1% of the participants and were replaced using the single imputation method. When a question was not asked to one or more companies, the responses were coded as missing data for those companies.

The confidence interval of correct compliance with malaria chemoprophylaxis was estimated by taking into account the clustered design of the study using the svy commands under STATA 9.0. Compliance with chemoprophylaxis was analysed as a dependant variable according to individual and company characteristics using a random effect mixed logistic regression model. The model was designed to take into account the intra-company correlations that could exist due to the sampling design (company effect as random effect). The logistic model was also adjusted using a generalized estimating equations (GEE) approach. Random effect and GEE regression models allow the estimation of company-specific and population-averaged effects, respectively [32].

First, a descriptive analysis of the independent variables was performed. A bivariate analysis was then conducted by entering each independent variable in a logistic regression model. Variables were retained for the multivariate analysis when their effect had a *p*-value less than 0.25 [33]. A backward stepwise selection procedure was applied to retain the significant (*p *< 0.05) independent variables and their interactions in the final model. The statistical quality of the final model was assessed by looking at the adequacy between observed and predicted probabilities of correct compliance.

## Results

### Study population

Of 2,901 eligible soldiers from 19 companies, 808 were excluded because they did not complete both questionnaires, leaving a sample of 2,093 subjects. The most common reason for not completing the questionnaires was the absence of the subject from the regiment at the time of the survey (because of holidays, training or a mission). Some soldiers who had planned to deploy to Africa and completed the first questionnaire did not travel and were replaced by others who had not completed the survey. Some soldiers returned to France before the rest of their company or were transferred to another regiment and were not present at the time of the second survey. The rate of refusal to participate in the study was lower than 5%.

The descriptive characteristics of the companies are summarized in Table 1. A total of 17 clinical malaria cases occurred during the mission among the 2,093 soldiers, which corresponds to an incidence rate of 0.81 cases per 100 soldier-missions (2.37 cases per 100 soldier-years).

**Table 1:** Characteristics of the companies

Comp	Country of the mission	Mission dates (MM/YY)		Type of the mission	Nb of soldiers	Nb of men	Age: median (25%-75% quantile)	Nb of soldiers with correct compliance	Nb of clinical malaria attacks
		Start	End						
1	Ivory Coast	02/04	06/04	Operation	144	143	26 (25 - 29)	51 (35.4%)	3
2	CAR	02/05	05/05	Operation	115	115	26 (23 - 29)	53 (46.1%)	2
3	Ivory Coast	02/05	05/05	Operation	108	108	22 (21 - 26)	70 (64.8%)	1
4	Ivory Coast	02/05	05/05	Operation	95	95	22 (20 - 24)	55 (57.9%)	0
5	Ivory Coast	02/05	05/05	Operation	73	72	23 (22 - 26)	38 (52.1%)	1
6	Senegal	06/05	09/05	Training	133	132	26 (22 - 30)	16 (12%)	0
7	Ivory Coast	06/08	09/05	Operation	84	84	25 (22 - 28.5)	54 (64.3%)	0
8	Ivory Coast	06/08	09/05	Operation	72	66	24 (22.5 - 28.5)	44 (61.1%)	1
9	Chad	10/05	01/06	Operation	134	130	24 (21 - 27)	85 (63.4%)	2
10	Senegal	02/06	05/06	Training	102	100	25 (22 - 28)	16 (15.7%)	0
11	Chad	02/06	05/06	Operation	83	78	25 (22 - 29)	44 (53.0%)	1
12	Gabon	07/06	11/06	Training	94	94	23.5 (21- 29)	9 (9.6%)	1
13	Gabon	07/06	11/06	Training	114	114	24 (21 - 29)	12 (10.5%)	2
14	Chad	06/06	09/06	Operation	189	185	24 (21 - 29)	130 (68.8%)	0
15	Gabon	12/06	04/07	Training	125	123	25 (22 - 30)	49 (39.2%)	0
16	Gabon	12/06	04/07	Training	135	133	24 (22 - 31)	38 (28.1%)	1
17	CAR	06/07	09/07	Operation	102	102	23 (20 - 29)	78 (76.5%)	0
18	Ivory Coast	06/07	09/07	Operation	94	92	22 (20 - 25)	72 (76.6%)	1
19	Ivory Coast	06/07	09/07	Operation	97	96	23 (21 - 28)	53 (54.6%)	1
Total		02/04	09/07		2093	2062	24 (21 - 28)	967 (46.2%)	17

Comp: Company; Nb: Number; CAR: Central African Republic

### Compliance with the chemoprophylaxis

Assessment of compliance level with chemoprophylaxis was based on the self-reported occurrences of daily medication doses missed during the mission. "No missed doses" was indicated by 967 out of 2,093 soldiers, or 46.2% "correct" compliance. Between companies, the lowest proportion of correct compliance was 9.6%, and the highest was 76.6%.

Among the other three responses corresponding to incorrect compliance, "less than one missed dose per month" was declared by 637 soldiers (30.4%); "one or more missed doses per month but less than one missed dose per week" was declared by 298 soldiers (14.2%); and "one or more missed doses per week" was declared by 191 soldiers (9.1%).

The frequency of correct compliance was calculated according tothe following: variables related to the mission, demographics and behavioral characteristics; the occurrence of particular events during the mission and the perception of risks; use of anti-mosquito protective measures; use of malaria chemoprophylaxis; and medical history and occurrence of medical events during the mission. These data are shown in Tables 2, 3, 4, 5 and 6, respectively.

**Table 2:** Variables related to the mission as well as the demographics and behavioral characteristics

	N	N-CC	Prevalence of CC			
			% (95% CI)	OR	95% CI	p value
Type of the mission						< 0.001
Training	703	140	19.9 (10.0 - 29.8)	1		
Military operation	1390	827	59.5 (51.8 - 67.2)	6.17	3.36 - 11.32	
Duration of the mission (mo)				0.81	0.61 - 1.07	0.1313
Age						0.2150
18 - 24 y.	1071	503	47.0 (34.9 - 59.1)	1		
25 y. and more	1022	464	45.4 (34.115 - 56.4)	1.11	0.94 - 1.30	
Gender						0.3667
Men	2062	953	46.2 (34.8 - 57.6)	1		
Women	31	14	45.2 (29.6 - 60.7)	0.74	0.38 - 1.4	
Rank						0.0329
JRs - NCOs	1610	728	45.2 (33.9 - 56.6)	1		
Os - WOs	483	239	49.5 (37.7 - 61.3)	1.22	1.02 - 1.47	
Favorite time for activities						0.0097
Morning type	531	252	47.5 (35.0 - 59.9)	1		
Intermediate type	1248	585	46.9 (35.3 - 58.5)	0.83	0.69 - 1.00	
Evening type	314	130	41.4 (30.1 - 52.7)	0.68	0.52 - 0.87	
Bedtime during the mission						0.0033
Before or at midnight	1607	778	48.4 (35.7 - 61.1)	1		
After midnight	342	138	40.4 (30.0 - 50.7)	0.69	0.55 - 0.86	
NA (Company 1)	144	51	35.4 (27.5 - 43.3)	0.57	0.09 - 3.56	
Previous overseas travel						0.1778
No	435	244	56.1 (44.7 - 67.5)	1		
Yes	1658	723	43.6 (32.3 - 54.9)	0.87	0.72 - 1.06	
Tobacco consumption						0.0420
Nonsmoker	934	451	48.3 (36.2 - 60.4)	1		
Smoker 1-20 cig/d.	903	406	45.0 (33.0 - 56.9)	0.86	0.73 - 1.02	
Smoker >20 cig/d.	256	110	43.0 (32.3 - 53.7)	0.75	0.58- 0.96	

N: Number of subjects; CC: Subjects with correct compliance; NA: Not Available

JRs - NCOs: junior ranks and noncommissioned officers; Os - WOs: officers and warrant officers; Cig/d.: cigarettes per day.

**Table 3:** Variables related to the occurrence of events during the mission and the perception of risks

	N	N-CC	Prevalence of CC			
			% (95% CI)	OR	95% CI	p value
Birth of a child						0.1168
No	2040	941	46.1 (35.0 - 57.3)	1		
Yes	53	26	49.1 (29.4 - 68.7)	1.49	0.91 - 2.46	
Death of a relative or a friend						0.5877
No	2004	928	46.3 (35.1 - 57.5)	1		
Yes	89	39	43.8 (27.1 - 60.6)	1.11	0.76 - 1.63	
Disease of a relative or a friend						0.9812
No	1996	926	46.4 (35.0 - 57.8)	1		
Yes	97	41	42.3 (30.3 - 54.2)	1	0.69 - 1.44	
Announcement of a separation or a divorce						0.2850
No	2048	943	46.0 (34.7 - 57.4)	1		
Yes	45	24	53.3 (32.4 - 74.3)	1.34	0.78 - 2.29	
Perception of a dangerous situation						0.2358
No	2041	947	46.4 (35.0 - 57.8)	1		
Yes	52	20	38.5 (23.8 - 53.1)	0.73	0.44 - 1.23	
Perception of personal malaria risk compared to other soldiers						0.4728
Inferior	280	132	47.1 (34.3 - 60.0)	1		
Equivalent	1388	644	46.4 (34.3 - 58.5)	1.04	0.82 - 1.32	
Superior	425	191	45.0 (33.1 - 56.8)	0.92	0.70 - 1.21	
Perception of the severity of malaria						0.0295
Not severe	254	136	53.5 (42.7 - 64.4)	1.3	1.02 - 1.66	
Mildly severe	1473	654	44.4 (32.4 - 56.4)	1		
Very severe	366	177	48.4 (35.1 - 61.6)	1.22	0.99 - 1.51	
Perceived mosquito attractiveness compared to other soldiers						0.0024
Inferior to the others	608	275	45.2 (32.1 - 58.3)	0.77	0.64 - 0.93	
Equivalent to the others	998	490	49.1 (38.3 - 59.9)	1		
Superior to the others	487	202	41.5 (29.0 - 53.9)	0.75	0.61 - 0.91	

N: Number of subjects; CC: Subjects with correct compliance

**Table 4:** Variables related to adherence to anti-mosquito protective measures

	N	N-CC	Prevalence of CC			
			% (95% CI)	OR	95% CI	p value
Condition of the received bed net						0.0502
Bad condition	736	338	45.9 (32.3 - 59.5)	1		
Good condition	1145	543	47.4 (36.0 - 58.9)	1.19	1.00 - 1.42	
NA: Company 1 or no net received	212	86	40.6 (25.9 - 55.2)	-	-	
Use of repellents before bedtime						0.0144
Not always	2019	919	45.5 (34.4 - 56.6)	1		
Always	74	48	64.9 (45.0 - 84.8)	1.73	1.11 - 2.67	
Use of insecticides for clothes before bedtime						0.0592
Not always	1922	894	46.5 (34.5 - 58.5)	1		
Always	27	22	81.5 (71.4 - 91.6)	2.46	1.15 - 5.28	
NA (Company 1)	144	51	35.4 (27.5 - 43.3)	0.62	0.10 - 3.80	
Long clothes worn before bedtime						0.0026
Not always	1025	384	37.5 (22.7 - 52.2)	1		
Always	1068	583	54.6 (46.4 - 62.8)	1.33	1.11 - 1.61	
Use of a bed net during sleep						< 0.0001
Not always	981	411	41.9 (26.1 - 57.7)	1		
Always	1112	566	50.0 (37.9 - 62.1)	1.57	1.28 - 1.94	
Systematic use of protective measures: long clothes, repellents, and bed net						< 0.0001
No one	653	249	38.1 (21.4 - 54.9)	1		
One of them	671	277	41.3 (26.8 - 55.8)	1.31	1.05 - 1.63	
Two or three of them	769	441	57.4 (48.0 - 66.7)	2.03	1.56 - 2.65	

N: Number of subjects; CC: Subjects with correct compliance; NA: Not Available

**Table 5:** Variables related to the chemoprophylaxis

	N	N-CC	Prevalence of CC			
			% (95% CI)	OR	95% CI	p value
Drug prescribed						0.0592
Chloroquine - proguanil	409	259	63.3 (55.3 - 71.4)	1		
Doxycycline	1675	701	41.9 (29.9 - 53.8)	0.65	0.34 - 1.26	
Mefloquine or other	9	7	77. 8 (46.9 - 100.0)	2.9	0.58 - 14.44	
Tolerance to the intake						0.1077
Rather bad to very bad	173	64	37.0 (18.4 - 55.6)	1		
Rather good	1010	442	43.8 (32.7 - 54.9)	1	0.74 - 1.35	
Very good	910	461	50.7 (39.7 - 61.7)	1.19	0.88 - 1.61	
Regularity of the day time of the intake						0.0419
Rather at the same time	1622	826	50.9 (40.1 - 61.8)	1		
Rather at different times	327	90	27.5 (14.9 - 40.2)	0.75	0.59 - 0.94	
NA (Company 1)	144	51	35.4 (27.5 - 43.3)	0.58	0.10 - 3.44	

N: Number of subjects; CC: Subjects with correct compliance; NA: Not Available

**Table 6:** Variables related to the medical history of infectious diseases or the occurrence of diseases during the mission

	N	N-CC	Prevalence of CC			
			% (95% CI)	OR	95% CI	p value
Medical history of malaria						0.0002
No	1870	902	48.2 (37.0 - 59.5)	1		
Yes	180	50	27.8 (17.2 - 38.4)	0.59	0.44 - 0.79	
Does not know	43	15	34.9 (18.6 - 51.1)	0.51	0.28 - 0.91	
Medical history of dengue fever						0.0625
No	1961	921	46.97 (35.7 - 58.3)	1		
Yes	83	26	31.32 (19.3 - 43.3)	0.66	0.44 - 1.00	
Does not know	49	20	40.82 (22.0 - 59.7)	0.69	0.40 - 1.16	
Occurrence of a disease during the mission						0.1223
No	1177	540	45.9 (34.2 - 57.6)	1		
Yes	916	427	46.6 (34.5 - 58.8)	0.88	0.74 - 1.04	
Occurrence of clinical malaria during the mission						0.0074
No	2076	965	46.5 (35.2 - 57.8)	1		
Yes	17	2	11.8 (3.6 - 27.1)	0.18	0.05 - 0.63	

N: Number of subjects; CC: Subjects with correct compliance

The variables retained in the final mixed multivariate logistic regression model are shown in Table 7. Correct compliance was significantly associated with the systematic use of one or more protective measures against mosquito bites, the perception of clinical malaria as potentially not severe or very severe (versus moderately severe), the birth of a child and the type of military operations (combat or training activities). Incorrect compliance was significantly associated with eveningness, a medical history of clinical malaria and a perceived mosquito attractiveness inferior or superior to the other soldiers. Compliance was not significantly associated with gender, age, rank or previous travels in malaria-endemic areas. The occurrence of clinical malaria during the mission was significantly associated with a lack of compliance as measured by the questionnaire (OR = 5.7, 95% CI: 1.6-20.2, *p *= 0.0074).

**Table 7:** Multivariate logistic regression analysis

	N	N-CC	RE		
			
			aOR	IC 95%	p-value
Type of the mission					< 0.0001
Training	703	140	1.00		
Military operation	1390	827	6.46	3.92 - 10.65	
Preferred time for activities					0.0805
Morning type	531	252	1.00		
Intermediate type	1248	585	0.83	0.65 - 1.05	
Evening type	314	530	0.69	0.50 - 0.96	
Bedtime during the mission					< 0.0001
Before or at midnight	1607	778	1.00		
After midnight	342	138	0.73	0.56 - 0.96	
Not Available (Company 1)	144	51	0.30	0.17 - 0.52	
Birth of a child					0.0421
No	2040	941	1.00		
Yes	53	26	1.99	1.02 - 3.88	
Perception of the severity of malaria					0.0171
Not severe	254	136	1.44	1.06 - 1.96	
Mildly severe	1473	654	1.00		
Very severe	366	177	1.31	1.00 - 1.71	
Systematic use of protective measures: long clothes, repellents, and bed net					0.0001
No one	653	249	1.00		
One of them	671	277	1.39	1.03 - 1.87	
Two or three of them	769	441	2.12	1.49 - 3.01	
Medical history of clinical malaria					< 0.0001
No	1870	902	1.00		
Yes	180	50	0.45	0.30 - 0.66	
Does not know	43	15	0.44	0.22 - 0.87	
Perceived mosquito attractiveness, compared to other soldiers					0.0029
Inferior to the others	608	275	0.71	0.56 - 0.89	
Equivalent to the others	998	490	1.00		
Superior to the others	487	202	0.71	0.55 - 0.91	
Random effect (*i.e. *company effect)					< 0.0001

N: Number of subjects; CC: Subjects with correct compliance; NA: Not Available

aOR: adjusted OR; RE: Random Effect Model

There was no significant interaction between independent variables. The company effect was significant in the final model. The OR estimates were similar for both the random effect and GEE regression models.

## Discussion

Many studies have been conducted in order to identify factors that influence compliance with medications, most of which focused on treatments to cure, reduce or delay complications and symptoms caused by chronic diseases. Only a few studies have assessed compliance in the context of chemoprophylaxis. Recently, the concept of adherence has supplanted compliance, as adherence implies that the patient agrees with the prescribed recommendations rather than passively obeying them (World Health Organization, 2003) [34]. However, in the present study, the concept of compliance was more suitable in the particular context of mandatory prophylactic measures. This work identified both collective and individual determinants of correct compliance with malaria chemoprophylaxis among French soldiers serving a short-term mission in intertropical Africa.

### Individual factors

The systematic use of protective measures against mosquito bites (including long-sleeved clothes and pants, repellents and bed net) was associated with correct compliance with chemoprophylaxis. Whereas previous studies showed no association [28] or even a competition [35] between the use of prophylactic measures, the present study showed that those who stated compliance with chemoprophylaxis also stated compliance with anti-mosquito measures, suggesting common determinants of these prophylactic behaviors.

Among life events, only the birth of a child during the mission was associated with correct compliance. This could be explained by an increase in the level of responsibility felt towards the newborn, a greater maturity among those in a position to father a child or internal pressure from the family [23,36], any of which could lead to a better awareness of the danger of malaria.

The "morning-type" soldiers, as determined by both the reduced Horne and Östberg morningness-eveningness questionnaire and by sleep/wake hours during the mission, were more likely to have correct compliance. Similarly, "evening-type" soldiers were less compliant with chemoprophylaxis. Eveningness has been associated with certain personality traits and dimensions (such as novelty-seeking, impulsivity, independent behaviour, risk taking, anti-conformism, lack of persistence and extraversion) [37-39] and is linked to both genetic factors [40] and environment [41]. Furthermore, morning-types are known to have a healthier lifestyle [42,43]. Thus, eveningness appears to be a risk factor for lack of compliance with malaria chemoprophylaxis, and it could be considered a target for focused interventions.

It has been shown that travellers who perceived themselves at high risk of becoming ill with malaria after returning home [26] or travellers to high-risk malaria-endemic areas who had correct risk perceptions [28] were more often compliant with chemoprophylaxis. The present study showed that subjects who either had a high or a low perception of the potential severity of clinical malaria were more often compliant compared to those who perceived it as mildly severe. This unexpected significant association persisted after adjusting for the other covariates and was explained by no interaction. The high level of compliance among those having a "very severe" perception confirmed the previous results. On the other hand, the "not severe" perception could be a consequence of feeling protected by one's correct compliance with an effective prophylactic measure. Indeed, perceptions and intentions must be considered together in behavioral studies [44].

Similarly, individuals who perceived themselves to be either more or less potent mosquito attractors than others were more compliant with chemoprophylaxis. This unexpected significant association also persisted after adjusting for the other covariates. Individuals who considered themselves less attractive to mosquitoes could be less compliant because of the perception of a lower risk of contracting malaria. In contrast, those who thought that they attracted mosquitoes more so than others were expected to be more compliant with anti-mosquito measures and could have believed that these anti-vectorial measures were sufficient to protect them. However, no significant interaction was found between the effects of the perceived mosquito attractiveness and compliance with anti-mosquito measures or chemoprophylaxis. Other determinants induced by the military setting such as fatalism or coping with danger by avoidance, denial or vulnerability could explain this unexpected association.

A previous medical history of malaria was associated with present lack of compliance. This suggests that having experienced an episode of clinical malaria was not a factor contributing by itself to the adoption of prophylactic behaviours [26]. Therefore, other arguments than the risk of contracting malaria should be used to convince these individuals to reach an optimal compliance with chemoprophylaxis.

The present study did not show any significant association between compliance and gender or age, although it has previously been shown that women and older travellers were more compliant [26,35,45]. However, the present work was conducted among French soldiers, where the proportion of women and older persons was small, leading to a lack of power to show such associations.

### Collective factors

Military operation-type missions were associated with correct compliance, independent of the country or the army corps. In such conditions, the soldiers were probably more closely supervised by the command, so they were submitted to a higher pressure to comply with the chemoprophylaxis. Indeed, the good health of the soldiers, including the prevention of malaria, was an important concern of the command because of the operational nature of the mission: the global health of the group had to be preserved for a military purpose [46]. Compliance with malaria chemoprophylaxis was also better among civilian travellers who were taking part in an organized trip compared to those traveling independently [35].

After taking all these factors into account, there was still a significant group effect; that is, significant behaviour determinants associated with the companies that were not identified in the present study. Moreover, the rate of compliance was very heterogeneous among companies, meaning probably that individual behaviours were not independent within a company. This unconscious or conscious imitation was not explained by the previous determinants. The lack of isolated soldier limited the possibility of studying the group effect. Compliance with anti-malarial prevention measures appears to be the end result of a complex mixing of the perception of risks and of protective/curative interventions, the acceptance of these measures and their application in spite of discomfort; qualitative approaches could be appropriate for exploring these complexities [47].

In this study, the compliance was measured based on a self-administered questionnaire. This method could overestimate the rate of correct compliance, as previously shown for anti-viral therapy [48] or malaria chemoprophylaxis [45]. A preliminary study with an electronic device (MEMS^®^: Medication Event Monitoring System) that records the date and time of bottle cap opening has been conducted to objectively assess the rates of compliance in the first company. This electronic monitoring is considered to be more accurate than questionnaires, diaries or counts of returned untaken tablets for the assessment of compliance [49,50]. If correct compliance is defined as 90% or more of daily prophylactic drug intake completed (using the MEMS^® ^as the reference), the answers to the questionnaire used in the present study had a 0.69 and 0.75 sensitivity and specificity, respectively, to the assessment of the correct compliance. These quality indexes were close to those previously found by others using only one question [49,51,52]. The questionnaire used in the present study was then considered appropriate for investigation of compliance. Indeed, the occurrence of clinical malaria during the mission was strongly associated with compliance as estimated by the questionnaire.

The results of the present study are applicable to French soldiers travelling for a short-term mission in intertropical Africa because the companies were representative of the different malaria-endemic areas to which they could be sent. The prevalence of correct compliance with malaria chemoprophylaxis among soldiers was close to those reported elsewhere among civilian travellers [4,28,35], and several determinants of compliance were similar to those identified among civilian travellers as well. Even if all the results of the present study could not be directly extrapolated to civilian travellers, they are useful for identifying key factors to improve compliance with malaria chemoprophylaxis. Indeed, the present study conducted on a large sample of travellers has shown collective and individual factors, including *a priori *determinants likeeveningness, which are associated with compliance. The identification of circumstances and profiles of persons at higher risk of lack of compliance would allow the implementation of specifically targeted strategies of health information, education, and communication, in order to improve compliance with malaria chemoprophylaxis and therefore its effectiveness.

## Competing interests

The authors declare that they have no competing interests.

## Authors' contributions

NR performed the statistical analysis and wrote the article. VM participated significantly in the data management. LO, EOP and GT participated significantly in the data management and the conception of the questionnaires. JG participated in the statistical analysis. BP, CTT and AB contributed significantly in the preparation of the study. CR conceived the study, took part in the analysis of the data and the discussion and wrote the article. The final version of the manuscript was seen and approved by all authors.
